# Prevalence of major electrocardiographic abnormalities in patients with hypertension in a primary care clinic in Hong Kong

**DOI:** 10.1186/s12872-022-02662-1

**Published:** 2022-05-18

**Authors:** Yuen Ying Tin, Lit Ping Chan, Jonathan Gabriel Sung, Shuk Yun Leung, Eric Ming Tung Hui, Maria Kwan Wa Leung

**Affiliations:** 1 Department of Family Medicine, Wong Siu Ching Family Medicine Centre, New Territories East Cluster, Hospital Authority, G/F, 1 Po Wu Lane, Tai Po, Hong Kong SAR China; 2 Department of Medicine and Geriatrics, Tuen Mun Hospital, Hong Kong SAR, China

**Keywords:** Hypertension, Electrocardiographic abnormalities, Primary care

## Abstract

**Background:**

Hypertension is strongly associated with cardiovascular events. Studies have shown that electrocardiographic (ECG) abnormalities were associated with increased risks for cardiovascular events. However local data is limited. The objectives of this study were: (1) to determine the prevalence of major electrocardiographic abnormalities in patients with hypertension in primary care in Hong Kong, and (2) to determine the association of major electrocardiographic abnormalities with patients’ socio-economical background, cardiovascular disease and cardiovascular risk factors.

**Methods:**

This was a cross-sectional study. Subjects were hypertensive patients aged between 18 and 80 who were enrolled in the Risk Assessment and Management Programme (RAMP) in a general outpatient clinic in Hong Kong. Outcome measures were prevalence of probable ischaemic heart disease (IHD), complete left bundle branch block (LBBB), left ventricular hypertrophy (LVH) and atrial fibrillation (AF) in patients with hypertension. The Pearson Chi-square test, independent t-test and Mantel–Haenszel test were used to measure the association between socioeconomic characteristics and cardiovascular risk factors, and ECG abnormalities.

**Results:**

504 hypertensive patients aged 18–80 were recruited in a general outpatient clinic. 6.3% had probable IHD, 0.4% had complete LBBB, 4.0% had LVH and 1.0% had AF. Probable IHD was associated with smoking (P = 0.032), hypercholesterolaemia (P = 0.037) and higher 10-year CV risk (P = 0.04). Complete LBBB was associated with smoking (P = 0.021) and hypercholesterolaemia (P = 0.022). LVH was associated with male gender (P = 0.001) and longer duration of hypertension (P = 0.035). AF was not significantly associated with any of the clinical or sociodemographic parameters.

**Conclusions:**

This study showed that a significant proportion of patients with hypertension at the primary care setting in Hong Kong had probable ischaemic heart disease, left ventricular hypertrophy and atrial fibrillation. This finding is consistent with both overseas data and historic data in Hong Kong. The detection of electrocardiographic abnormalities is helpful in hypertension management by improving risk stratification.

**Supplementary Information:**

The online version contains supplementary material available at 10.1186/s12872-022-02662-1.

## Background

Hypertension is one of the leading causes for global mortality. It is estimated that 1.13 billion people worldwide are diagnosed with hypertension [1]. The latest Population Health Survey conducted by the Department of Health in Hong Kong reported that around 17.8% of the population aged 15 or above were diagnosed of hypertension [2].

Hypertension is strongly associated with cardiovascular (CV) events, especially ischaemic heart disease and stroke [3]. It was recommended by the *2018 ESH/ESC Guidelines for the management of arterial hypertension* that a 12-lead electrocardiogram (ECG) should be performed on all hypertensive patients as part of routine assessment [4]. ECG is the most readily available investigation at primary care setting for the detection of cardiac abnormalities. It is non-invasive with virtually no risk posed to the patients. The results can be interpreted rapidly by the attending family physician with little inter-observers’ variability.

Several studies have shown that major and minor abnormalities on resting 12-lead ECG were consistently associated with increased risks for cardiovascular events. A population based cohort study from the Health ABC Study in the United States reported such association among adults aged 70 to 79 [5]. Another cross-sectional study indicated the independent association between ECG abnormalities and mortality from coronary heart disease and all cardiovascular diseases in middle-aged United States men and women [6]. This association is also valid in different ethnicities [7–9].

The detection of electrocardiographic abnormalities is helpful in hypertension management by improving risk stratification. In a United States national sample, it was shown that 28% had ≥ 1 major ECG abnormality and the prevalence was greater among those older than 65 years of age [10]. In another large retrospective study, electrocardiographic abnormalities were observed in more than 50% of patients with hypertension attending primary care centres in Brazil [11]. In Hong Kong, a study on a subsample of a territory-wide health survey for elderly subjects conducted in 1993 reported that the prevalence of electrographic abnormalities was 5.8% in men and 6.5% for women of age 70 or older. The findings were comparable to elderly Caucasian populations [12]. However there is limited evidence regarding prevalence of electrocardiographic abnormalities in primary care setting in Hong Kong.

## Methods

### Aim of study

The primary objective of this study was to determine the prevalence of major electrocardiographic abnormalities in patients with hypertension in primary care in Hong Kong. This study mainly focused on hypertensive heart diseases, namely probable ischaemic heart disease (IHD), complete left bundle branch block (LBBB), left ventricular hypertrophy (LVH) and atrial fibrillation (AF). The secondary objective was to evaluate the association between major electrocardiographic abnormalities and socio-economical background, underlying cardiovascular disease and other cardiovascular risk factors in patients with hypertension.

### Study design and subjects

This was a cross-sectional study, which was carried out in a government general outpatient clinic that serves a population of around 300,000 in Hong Kong. According to the Primary Care Service Statistics Portfolio by the Hospital Authority of Hong Kong [13], the disease profile of the patients from this clinic is similar to those in other general outpatient clinics in Hong Kong. Around 60% of our patients have chronic illness. In particular 50% of our patients have hypertension, which was comparable to other public general outpatient clinic in Hong Kong. Patients with hypertension who were enrolled in the Risk Assessment and Management Programme for Hypertension (RAMP-HT) were recruited. Inclusion criteria were hypertensive patients fulfilling the criteria of hypertension by JNC 7 [14], aged between 18 to 80 years old and were either on lifestyle modification only or receiving antihypertensive medications. Patients who were diabetic, bed bound, more than 80 years of age, with known ischaemic heart disease, incomplete or missing data, or incapable of giving free informed consent were excluded. These were in line with the exclusion criteria of the RAMP programme.

### Measurements and outcomes

The duration of the study was 4 months, from December 2017 to March 2018. All hypertensive patients in the RAMP-HT were arranged to have blood tests including fasting blood glucose, lipid profile, renal function test and estimated Glomerular Filtration Rate by MDRD. Basic parameters including blood pressure, pulse rate and body mass index (BMI) were measured according to standard protocol. Urine protein was measured by urine albustix strip. ECG was routinely performed. Patients who agreed to participate in the study were invited to fill in a questionnaire (Additional file 1: Appendix A) on their socio-demographic data, duration of hypertension, comorbidities, subjective well-being and symptom of angina (based on the WHO Rose angina questionnaire with Chinese translation performed by the investigators of this study) [15, 16]. Clinical information was verified with patient’s case notes via Clinical Management System (CMS). Illegible or missing information was clarified by nurses.

The 10-year CVD risk was calculated using the JBS Equation 2005 based on age, gender, smoking status, total cholesterol, HDL cholesterol and pre-treatment systolic blood pressure (160 mmHg if unknown) [17].

Each ECG was reviewed by two trained family physicians, who classified the ECG as (1) normal; (2) probable ischaemic heart disease (major Q or QS wave); (3) complete left bundle branch block; (4) left ventricular hypertrophy; (5) atrial fibrillation and (6) other according to the Minnesota Code [18] (Additional file 2: Appendix B). Minnesota Code is the most widely used coding system for ECG in population based studies [19]. It provides a classification of electrocardiographic morphology on the basis of rigid criteria and allows objective comparison of ECG findings. Discordant results were adjudicated by a cardiologist.

### Statistical methods

Data was analysed using IBM SPSS Statistics 22. The Pearson Chi-square test was used to measure the association between socioeconomic characteristics and cardiovascular risk factors (including sex, smoking status, mobility, occupation, marital status, financial aid, housing, impaired fasting glucose, hypercholesterolemia, microalbuminuria and stroke) and electrocardiographic abnormalities. Independent t-test was used for measuring the association between age, renal impairment and BMI and electrocardiographic abnormalities, while Mantel–Haenszel test was used for 10-year cardiovascular risk, duration of hypertension, education and income. A p value of < 0.05 was considered statistically significant.

## Results

504 patients were recruited for the analysis. Of them 270 (53.6%) were male and 234 (46.5%) were female (Table 1). 91.3% of the recruited patients were aged 50 or above, with 44.6% were between 60 to 69 years of age. 27.4% of patients were either current smokers or ex-smokers. The mean office systolic and diastolic blood pressures at the time of recruitment were 135.9 ± 16.3 mmHg and 79.5 ± 10.5 mmHg respectively.

**Table 1:** Sociodemographic and clinical characteristics of patients (N = 504)

	Number (%)	Mean ± SD
Sex		
Male	270 (53.6)	
Female	234 (46.4)	
Age (years)		61.6 ± 9.1
20–39	15 (3.0)	
40–49	29 (5.8)	
50–59	145 (28.8)	
60–69	225 (44.6)	
70–79	90 (17.9)	
Smoking status		
History of smoking	138 (27.4)	
Non-smoker	366 (72.6)	
Blood pressure (mmHg)		
Systolic		135.9 ± 16.3
Diastolic		79.5 ± 10.5
Duration of HT (year)		
Less than 1	163 (32.4)	
1–5	170 (33.7)	
6–10	82 (16.3)	
11–15	49 (9.7)	
16–20	20 (4.0)	
20–25	15 (3.0)	
More than 25	5 (1.0)	
Fasting glucose (mmol/L)		
< 5.6	353 (70)	
5.7 – 6.9	151 (30)	
Hypercholesterolemia (LDL ≥ 4.1 mmol/L or on lipid-lowering drug)		
Yes	140 (27.8)	
No	364 (72.2)	
Estimated glomerular filtration rate (ml/min/1.73m^2^)		
< 30	0 (0)	
30–60	45 (8.9)	
60–90	323 (64.1)	
> 90	136 (27.0)	
Urine albustix		
+	32 (6.3)	
−	472 (93.7)	
BMI (kg/m^2^)		
≤ 18.5	6 (1.2)	
18.6–22.9	78 (15.5)	
23–24.9	131 (26.0)	
≥ 25	289 (57.3)	
10 Yr CV risk		
Low (< 10%)	46 (9.1)	
Medium (10–20%)	200 (39.7)	
High (> 20%)	258 (51.2)	
Other Medical Illness other than HT	*Some have > 1 comorbidities	
Nil	366 (72.6)	
Cardiac disease	8 (1.6)	
Ischaemic heart disease	0 (0)	
Arrhythmia	4 (0.8)	
Valvular disease	4 (0.8)	
Heart failure	0 (0)	
Stroke	26 (5.2)	
PVD	0 (0)	
Kidney disease	6 (1.2)	
Fatty liver	30 (6.0)	
COPD	3 (0.6)	
Other	70 (13.9)	
Rose Angina Questionnaire		
No angina	479 (95.3)	
Grade 1 angina	7 (1.4)	
Grade 2 angina	18 (3.6)	
Mobility		
Unaided	486 (96.4)	
Walk with stick	15 (3.0)	
Wheelchair	3 (0.6)	
Occupation		
Full time	170 (33.7)	
Part time	50 (9.9)	
Retired	171 (33.9)	
Housewife	103 (20.4)	
Unemployed	10 (2.0)	
Education		
No formal education	34 (6.7)	
Primary school	171 (33.9)	
Up to Form 3	140 (27.8)	
Up to Form 7	122 (24.2)	
Diploma	19 (3.8)	
University degree or above	18 (3.6)	
Marital status		
Married	439 (87.1)	
Single	23 (4.6)	
Separated	1 (0.2)	
Divorced	25 (5.0)	
Widowed	16 (3.2)	
Co-habitat	0 (0.0)	
CSSA		
Yes	58 (11.5)	
No	446 (88.5)	
Monthly salary (HK$)		
Less than 5000	245 (48.6)	
5001–10,000	94 (18.7)	
10,001–15,000	62 (12.3)	
15,001–20,000	45 (8.9)	
20,001–25,000	25 (5.0)	
25,001–30,000	11 (2.2)	
30,001–35,000	9 (1.8)	
35,001–40,000	6 (1.2)	
40,000 above	7 (1.4)	
Housing		
Private property	151 (30.0)	
Home ownership scheme	122 (24.2)	
Public housing	170 (33.7)	
Rental	45 (8.9)	
Other	16 (3.2)	

Overall 66.5% of the patients had normal ECG (Table 2). 6.3% had probable ischaemic changes on ECG. 0.4% patients had complete left bundle branch block, and these patients were not included in the group of probable IHD. ECG features of left ventricular hypertrophy, a form of asymptomatic hypertension mediated organ damage, were detected in 4.0% of the patients. 1.0% were found to have atrial fibrillation, an important co-morbidity in hypertensive patients. 21.8% had other minor ECG abnormalities inculding sinus bradycardia, first degree heart block and premature atrial complex. 0.6% patients had multiple abnormalities (sinus bradycardia with first degree heart block).

**Table 2:** Prevalence of electrocardiographic abnormalities (N = 504)

Electrocardiographic findings	Number (%)
Normal	335 (66.5)
Probable IHD	32 (6.3)
Complete LBBB	2 (0.4)
LVH	20 (4.0)
AF	5 (1.0)
other	110 (21.8)

Tables 3 demonstrated the association of major ECG abnormalities and sociodemographic and clinical characteristics. Probable IHD was significantly associated with history of smoking (P = 0.032), hypercholesterolemia (P = 0.037) and higher 10-year CV risk (P = 0.04). It tended to be associated with patients on Comprehensive Social Security Assistance (CSSA) but was not statistically significant. Complete LBBB was found to be significantly associated with history of smoking (P = 0.021) and hypercholesterolaemia (P = 0.022). LVH was significantly associated with male gender (P = 0.001) and longer duration of hypertension (P = 0.035), with prevalence of 1.8% for patients with 1–5 years’ history of hypertension compared to 13.3% for 20–25 years. It also tended to associate with higher 10-year CVD risk (> 20%) but was not statistically significant. AF was not significantly associated with any of the clinical or sociodemographic parameters.

**Table 3:** Association of major ECG abnormalities and sociodemographic and clinical characteristics (N = 504)

		Probable IHD (n = 32)		Complete LBBB (n = 2)		LVH (n = 20)		AF (n = 5)	
Sex	N =								
Male	270	20	7.4%	0	0.0%	18	6.7%	3	1.1%
Female	234	12	5.1%	2	0.9%	2	0.9%	2	0.9%
p value			0.290		0.128		0.001		0.772
Mean age		62.6	64.5	66.3	69				
p value		0.557	0.291	0.119	0.186				
Smoking									
Non-smoker	366	18	5.5%	0	0.0%	14	3.8%	3	0.8%
History of smoking	138	14	8.7%	2	1.4%	6	4.3%	2	1.4%
p value			0.032		0.021		0.789		0.525
FG									
< 5.6	332	19	5.7%	1	0.3%	12	3.6%	2	0.6%
5.6 – 6.9	172	13	7.6%	1	0.6%	8	4.7%	3	1.7%
p value			0.423		0.635		0.572		0.220
Hypercholesterolemia (LDL ≥ 4.1 or on lipid-lowering drug)									
Yes	140	14	10.0%	2	1.4%	6	4.3%	2	1.4%
No	364	18	4.9%	0	0.0%	14	3.8%	3	0.8%
p value			0.037		0.022		0.821		0.540
eGFR									
< 30	0	0		0		0		0	
30–60	45	0	0.0%	0	0.0%	4	8.9%	0	0.0%
60–90	323	22	6.8%	0	0.0%	16	5.0%	5	1.5%
> 90	136	10	7.4%	2	1.5%	0	0.0%	0	0.0%
p value			0.239		0.682		0.143		0.515
Urine albustix									
+	32	2	6.3%	0	0.0%	0	0.0%	0	0.0%
−	472	30	6.4%	2	0.4%	20	4.2%	5	1.1%
p value			0.987		0.712		0.406		0.558
BMI									
≤ 18.5	6	0	0.0%	0	0.0%	0	0.0%	0	0.0%
18.6–22.9	78	0	0.0%	2	2.6%	4	5.1%	2	2.6%
23–24.9	131	8	6.1%	0	0.0%	6	4.6%	0	0.0%
≥ 25	289	24	8.3%	0	0.0%	10	3.5%	3	1.0%
p value			0.198		0.482		0.344		0.993
10 Yr CV risk									
Low (< 10%)	46	0	0.0%	0	0.0%	0	0.0%	0	0.0%
Medium (10–20%)	200	8	4.0%	1	0.5%	4	2.0%	3	1.5%
High (> 20%)	258	24	9.3%	1	0.4%	16	6.2%	2	0.8%
p value			0.04		0.86		0.06		0.94
Duration of HT (year)									
Less than 1	163	12	7.4%	2	1.4%	6	3.7%	1	0.7%
1–5	170	10	5.9%	0	0.0%	3	1.8%	3	1.8%
6–10	82	6	7.3%	0	0.0%	3	3.7%	0	0.0%
11–15	49	2	4.1%	0	0.0%	4	8.2%	1	2.0%
16–20	20	2	10.0%	0	0.0%	2	10.0%	0	0.0%
20–25	15	0	0.0%	0	0.0%	2	13.3%	0	0.0%
More than 25	5	0	0.0%	0	0.0%	0	0.0%	0	0.0%
p value			0.399		0.19		0.035		0.60
Other CVD									
None	466	32	6.9%	2	0.4%	18	3.9%	4	0.9%
Stroke/TIA	36	0	0.0%	0	0.0%	2	5.6%	1	2.8%
CKD	0	0	0.0%	0	0.0%	0	0.0%	0	0.0%
PVD	0	0	0.0%	0	0.0%	0	0.0%	0	0.0%
Retinopathy	2	0	0.0%	0	0.0%	0	0.0%	0	0.0%
p value			0.26		0.694		0.723		0.262
Mobility									
Unaided	486	31	6.4%	2	0.4%	20	4.1%	4	0.8%
Walk with stick	15	3	20.0%	0	0.0%	0	0.0%	1	6.7%
Wheelchair	3	0	0.0%	0	0.0%	0	0.0%	0	0.0%
p value			0.082		0.994		0.680		0.166
Occupation									
Full time	170	12	7.1%	1	0.6%	4	2.4%	1	0.6%
Part time	50	1	2.0%	0	0.0%	2	4.0%	1	2.0%
Retired	171	11	6.4%	1	0.6%	12	8.2%	3	1.8%
Housewife	103	8	7.8%	0	0.0%	2	0.0%	0	0.0%
Unemployed	10	0	0.0%	0	0.0%	0	0.0%	0	0.0%
p value			0.597		0.966		0.146		0.711
Education									
No formal education	35	3	8.6%	0	0.0%	0	0.0%	0	0.0%
Primary School	171	14	8.2%	1	0.6%	12	7.0%	2	1.2%
Up to Form 3	140	7	5.0%	1	0.7%	4	2.9%	1	0.7%
Up to Form 7	122	6	4.9%	0	0.0%	4	3.3%	2	1.6%
Diploma	19	1	5.3%	0	0.0%	0	0.0%	0	0.0%
University Degree or above	18	1	5.6%	0	0.0%	0	0.0%	0	0.0%
p value			0.157		0.582		0.168		0.065
Marital Status									
Married	439	28	6.4%	1	0.2%	20	4.6%	4	0.9%
Single	23	0	0.0%	0	0.0%	0	0.0%	0	0.0%
Separated	1	0	0.0%	0	0.0%	0	0.0%	0	0.0%
Divorced	25	4	16.0%	1	4.0%	0	0.0%	0	0.0%
Widowed	16	0	0.0%	0	0.0%	0	0.0%	1	6.3%
Co-habitat	0	0		0		0		0	
p value			0.527		0.069		0.544		0.285
CSSA									
Yes	58	7	12.1%	0	0.0%	1	1.7%	1	1.7%
No	446	25	5.6%	2	0.4%	19	4.3%	4	0.9%
P value			0.064		0.606		0.341		0.561
Monthly salary (HK$)									
Less than 5000	245	21	8.6%	1	0.4%	10	4.1%	4	1.6%
5001–10,000	94	3	3.2%	1	1.1%	3	3.2%	1	1.1%
10,001–15,000	62	4	6.5%	0	0.0%	2	1.6%	0	0.0%
15,001–20,000	45	1	2.2%	0	0.0%	2	4.4%	0	0.0%
20,001–25,000	25	1	4.0%	0	0.0%	0	0.0%	0	0.0%
25,001–30,000	11	0	0.0%	0	0.0%	1	9.1%	0	0.0%
30,001–35,000	9	1	11.1%	0	0.0%	0	0.0%	0	0.0%
35,001–40,000	6	0	0.0%	0	0.0%	1	16.7%	0	0.0%
40,000 above	7	1	14.3%	0	0.0%	1	14.3%	0	0.0%
p value			0.41		0.64		0.47		0.12
Housing									
Private property	151	10	6.6%	0	0.0%	4	2.6%	2	1.3%
Home Ownership Scheme	122	7	5.7%	0	0.0%	6	4.9%	0	0.0%
Public Housing	170	13	7.6%	2	1.2%	5	2.9%	1	0.6%
Rental	45	2	4.4%	0	0.0%	3	6.7%	0	0.0%
Other	16	0	0.0%	0	0.0%	2	12.5%	2	12.5%
P value			0.747		0.562		0.252		0.099

Mean age was similar among patients with or without ECG abnormalities. Fasting glucose, BMI and renal function were not shown to be a significant risk factor for patients with the concerned ECG abnormalities. The risks of probable IHD, complete LBBB, LVH and AF are not shown to be significantly different in hypertensive patients with varying degree of mobility, occupation, education level, marital status monthly salary and housing. There were no significant associations between ECG abnormalities and blood pressure levels (Table 4).

**Table 4:** Association of ECG abnormalities with median BP

	Median SBP (mmHg)	Median DBP (mmHg)
IHD	136	82
Without IHD	135	79
p value	0.977	0.204
LBBB	126	72
Without LBBB	135	80
p value	0.528	0.479
LVH	133	75
Without LVH	135	80
p value	0.887	0.749
AF	127	74
Without AF	135	80
p value	0.9	1.0

Among the patients with probable IHD on ECG, one had grade 2 angina according to the WHO Angina Questionnaire.

## Discussion

This study examined the prevalence of cardiovascular conditions, in the form of electrocardiographic abnormalities, in hypertensive patients under primary care. Hypertension is one of the most important cardiovascular risk factors, based on its high prevalence in the community and strong association with serious cardiovascular conditions. However, the care for hypertension should take way beyond the blood pressure values alone as the only determinant in the clinical decision on treatment. Studies have shown that only a minority of the hypertensive population exhibits elevated blood pressure alone, with most patients having other cardiovascular risk factors and relevant socioeconomic background as well [4]. On the other hand, each of these factors might potentiate each other in the development of cardiovascular disease. It is therefore essential to study the hypertensive population in a comprehensive manner to aid the management of, and more importantly prevention of cardiovascular diseases.

This study showed that 69% of the patients had normal ECG, which was expected given the primary care setting under which these patients were recruited. 6.4% of the hypertensive population in the primary care setting had probable ischaemic heart disease based on ECG findings, while 4.0% had evidence of left ventricular hypertrophy on ECG. These findings were comparable to other similar studies. A population-based study in Hong Kong published in 1993 showed a prevalence of probable ischaemic heart disease of 5.8% for men and 6.5% for women of age over 70 years in a subsample of 197 subjects from a health survey for the elderlies [12]. The “Leiden 85-plus study” from the Netherlands revealed that among the population aged 85 years, 9% had probable ischaemic heart disease [20]. Furberg et al. from the Cardiovascular Health Study in the US reported that 5.2% of the population aged 65 years or above had major Q/QA waves, with over half of the cases being without a past myocardial infarction [21]. These findings were consistent with our results, showing that over the years there remained a significant proportion of the hypertensive population having probable ischaemic heart disease. Baseline ECG would allow early identification of IHD and hence further management such as cardiovascular risk factors modification. Further clinical audit could be done on this topic.

According to our findings, the prevalence of atrial fibrillation, an important risk factor for stroke, was 1.0%. This was comparable to a local study published in 2017 [22]. Given the age of these patients, together with history of hypertension, the annual stroke risk could be as high as 3–4% depending on the gender [23]. A screening ECG for this group of patients could lead to the prompt prescription of anticoagulation for suitable candidates and therefore a significant decrease in the stroke risk. The impact could hardly be overstated given the serious morbidity and mortality of cardioembolic stroke associated with atrial fibrillation.

Duration of hypertension correlated with the risk of LVH ECG, as patients with longer history of hypertension were more likely to have features of LVH on ECG. A cohort study involving 4290 patients conducted in Italy revealed similar finding. Patients with LVH had significantly longer duration of hypertension of 6.0 ± 6.5 years compared to 4.8 ± 5.9 years in patient without LVH (p < 0.0001) [24]. This is consistent with the common understanding that left ventricular myocytes hypertrophy in order to adapt to the increased afterload in patients with chronic hypertension [25, 26]. On the other hand, no significant associations were found between ECG abnormalities and blood pressure levels. Since the blood pressure used for analysis was only a single office measurement thus might not reflect patients’ ambulatory blood pressure control.

In Hong Kong, primary care service is provided by both government and private sectors. According to a survey conducted by the Chinese University of Hong Kong on 1000 adults in Hong Kong, among those with chronic conditions, 23.5% identified public general outpatient clinic as their main provider for chronic disease management. 13.6% identified a private GP and about half identified a government-operated specialist outpatient clinic as their main providers of chronic disease management. ECG has to be done for all newly diagnosed hypertension. Although this practice is done in our clinic routinely, it may not be the same in the private sector. Therefore it is vital to remind primary care physicians to perform baseline ECG on newly diagnosed hypertensive patients [27].

### Limitation

This study had several limitations. Firstly, similar to other population-based registries, the interpretation of this study is limited by the inherent weakness of registry data. For instance, our study showed that hypertensive patients on CSSA tended to have higher risk of probable IHD, although it was not statistically significant. However, the interpretation of this finding is limited by the absence of temporal information in registry data and hence a causal relationship could not be concluded. Secondly, there could be reporting bias as the socioeconomic information volunteered by the subjects could not be verified and hence solely based on the subjects’ own account of their history. However, as patients have been guaranteed on the confidentiality of their personal information during consent and patients unfit for consent have been excluded, their information should generally be trustworthy. Finally, as this study was carried out in primary care setting, the subjects were a highly selected group of patients and might not reflect the whole population. For example, nephropathy is an important complication of chronic hypertension. However, essentially all patients with significant renal impairment have been referred to tertiary centres for follow up and therefore would not have been included in this study. Moreover, patients with diabetes mellitus have also been excluded from this RAMP-HT programme through which our subjects were recruited. Despite all these limitations, this study could provide a fair reflection of the patients currently under care in our primary care system.

## Conclusions

This study showed that a significant proportion of the patients with hypertension at the primary care setting in Hong Kong had probable ischaemic heart disease, left ventricular hypertrophy and atrial fibrillation. This finding is consistent with both overseas data and historic data in Hong Kong. Smoking and hypercholesterolemia were significantly associated with probable IHD and complete LBBB. Male gender and longer duration of hypertension were significantly associated with LVH. Early diagnosis by ECG could lead to prompt and timely treatment of these conditions. Currently, ECG is performed for each hypertensive patient who has newly joined RAMP-HT screening programme in our practice. Further study could look into the need of repeating ECG in subsequent complication screening and the relevant impact on their prognosis.

## Supplementary Information

**Additional file 1. Appendix A**: Minnesota code.

**Additional file 2. Appendix B**: Questionnaire.

## Data Availability

The datasets used and/or analyzed during the current study are not publicly available as the data was collected directly from patient or patient’s medical records, which should be kept strictly confidential according to study protocol. However the datasets are available from the corresponding author on reasonable request.

## Abbreviations

CV: Cardiovascular
ECG: Electrocardiogram
IHD: Ischaemic heart disease
LBBB: Left bundle branch block
LVH: Left ventricular hypertrophy
AF: Atrial fibrillation
RAMP: Risk Assessment and Management Programme
BMI: Body mass index
CMS: Clinical Management System
eGFR: Estimated glomerular filtration rate
CSSA: Comprehensive Social Security Assistance
