# Supplementary material for: Prevalence of major electrocardiographic abnormalities in patients with hypertension in a primary care clinic in Hong Kong
Source: BMC Cardiovasc Disord. 2022 May 18;22:225. doi: 10.1186/s12872-022-02662-1 (PMC9118877; doi:10.1186/s12872-022-02662-1)
Supplement: Supplementary file 1 — Additional file 1. Appendix A: Minnesota code. [file 12872_2022_2662_MOESM1_ESM.docx]

**Prevalence of Major Electrocardiographic Abnormalities in Patients with Hypertension in a Primary Care Clinic in Hong Kong**

**Appendix A – Minnesota Code**

Note that only the Minnesota Code for probable ischaemic heart disease (major Q or QS wave), complete left bundle branch block, left ventricular hypertrophy and atrial fibrillation are listed here.

1. Probable ischaemic heart disease

| **Q-QS Waves (1-Codes)** | |
| --- | --- |
| Anterolateral Site (Leads I, aVL, V6) | |
| 1.1.1 | Q/R amplitude ratio >= 1/3, plus Q duration >= 0.03 sec in lead I or V6. |
| 1.1.2 | Q duration >= 0.04 sec in lead I or V6. |
| 1.1.3 | Q duration >= 0.04 sec, plus R amplitude >=3mm in lead aVL. |
| 1.2.1 | Q/R amplitude ratio >= 1/3, plus Q duration >= 0.02 sec and < 0.03 sec in lead I or V6. |
| 1.2.2 | Q duration >= 0.03 sec and < 0.04 sec in lead I or V6. |
| 1.2.3 | QS pattern in lead I. Do not code in the presence of 7-1-1. |
| Posterior (inferior) Site (Leads II, III, aVF) | |
| 1.1.1 | Q/R amplitude ratio >= 1/3, plus Q duration >= 0.03 sec in lead II. |
| 1.1.2 | Q duration >= 0.04 sec in lead II. |
| 1.1.4 | Q duration >= 0.05 sec in lead III, plus a Q-wave amplitude > 1.0 mm in the majority of beats in lead aVF. |
| 1.1.5 | Q duration >= 0.05 sec in lead aVF. |
| 1.2.1 | Q/R amplitude ratio >= 1/3, plus Q duration >= 0.02 sec and < 0.03 sec in lead II. |
| 1.2.2 | Q duration >= 0.03 sec and < 0.04 sec in lead II. |
| 1.2.3 | QS pattern in lead II. Do not code in the presence of 7-1-1. |
| 1.2.4 | Q duration >= 0.04 sec and < 0.05 sec in lead III, plus a Q-wave >= 1.0 mm amplitude in the majority of beats in aVF. |
| 1.2.5 | Q duration >= 0.04 sec and < 0.05 sec in lead aVF. |
| Anterior Site (Leads V1, V2, V3, V4, V5) | |
| 1.1.1 | Q/R amplitude ratio >= 1/3, plus Q duration >= 0.03 sec in any of leads V2, V3, V4, V5. |
| 1.1.2 | Q duration >= 0.04 sec in any of leads V1, V2, V3, V4, V5. |
| 1.1.6 | QS pattern when initial R-wave is present in adjacent lead to the right on the chest, in any of leads V2, V3, V4 V5, V6. |
| 1.1.7 | QS pattern in all of leads V1-V4 or V1-V5. |
| 1.2.1 | Q/R amplitude ratio >= 1/3, plus Q duration >= 0.02 sec and < 0.03 sec in any of leads V2, V3, V4, V5. |
| 1.2.2 | Q duration >= 0.03 sec and < 0.04 sec in any of leads V2, V3, V4, V5. |
| 1.2.7 | QS pattern in all of leads V1, V2, and V3. (Do not code in the presence of 7-1-1.) |

1. Complete left bundle branch block

| **Intraventricular Conduction Defects (7-Codes)** | |
| --- | --- |
| 7.1.1 | (Do not code in presence of 6-1, 6-4-1, 6-8, 8-2-1 or 8-2-2.) QRS duration >= 0.12 sec in a majority of beats (of the same QRS pattern) in any of leads I, II, III, aVL, aVF, plus R peak duration >= 0.06 sec in a majority of beats (of the same QRS pattern) in any of leads I, II, aVL, V5. V6. (7-1-1 suppresses 1-2-3, 1-2-7, 1-3-2, 1-3-8, 1-3-6 and all 2, 3, 4, 5, 9-2, 9-4, 9-5 codes. If any other codable Q-wave coexists with the LBBB pattern, code the Q and diminish the 7-1-1 code to a 7-4 code.) |

1. Left ventricular hypertrophy

| **High R-Waves (3-Codes)** | |
| --- | --- |
| 3.1 | Left. R amplitude > 26 mm in either V5 or V6, or R amplitude > 20.0 mm in any of leads I, II, m, aVF, or R amplitude > 12.0 mm in lead aVL measured only on second to last complete normal beat. |

1. Atrial fibrillation

| **Arrhythmias (8-Codes)** | |
| --- | --- |
| 8.3.1 | Atrial fibrillation (persistent). |
| 8.3.2 | Atrial flutter (persistent). |
