# Supplementary material for: Prevalence of major electrocardiographic abnormalities in patients with hypertension in a primary care clinic in Hong Kong
Source: BMC Cardiovasc Disord. 2022 May 18;22:225. doi: 10.1186/s12872-022-02662-1 (PMC9118877; doi:10.1186/s12872-022-02662-1)
Supplement: Supplementary file 2 — Additional file 2. Appendix B: Questionnaire. [file 12872_2022_2662_MOESM2_ESM.docx]

**Prevalence of Major Electrocardiographic Abnormalities in Patients with Hypertension in a Primary Care Clinic in Hong Kong**

**Appendix B – Questionnaire**

**Questionnaire – Prevalence of Major Electrocardiographic Abnormalities in Patients with Hypertension in Primary Care in Hong Kong**

1) How long have you been suffering from Hypertension?

1 Less than 1 year 2 1-5 years 3 6-10 years ≤4 11-15 years

5 16-20 years 6 20-25 years ≤7 More than 25 years ≤8 Not sure

2) Do you have other medical illness other than Hypertension?

1 No

2 Cardiac disease:

2a Ischaemic Heart Disease 　2b Arrhythmia 　2c Valvular Disease

2d Heart Failure

3 Stroke

4 Peripheral Vascular Disease

5 Kidney Disease

6 Fatty Liver

7 Chronic Obstructive Pulmonary Disease

8 Other: _____________

3) How would you comment on your current health condition?

1 Very Good 2 Good 3 Fair 4 Bad ≤5 Very Bad

4) Chest pain or chest discomfort

a) Have you ever had any pain or discomfort in your chest?

Yes No

b) Do you get this pain or discomfort when you walk uphill or hurry?

Yes No

c) Do you get it when you walk at an ordinary pace on the level?

Yes No

d) When you get any pain or discomfort in your chest, what do you do?

Stop Slow down Continue at the same pace

e) Does it go away when you stand still?

Yes No

f) How soon?

10 minutes or less More than 10 minutes

g) Have you ever consulted a doctor for the above condition?

Yes No

5) Have you ever had a electrocardiogram (ECG) before? (Excluding ECG today)

1 Yes ≤2 No

======================================================

1) Mobility: 1 Unaided ≤2 Walk with stick ≤3 Wheelchair

2) Occupation: 1 Full time 2 Part time ≤3 Retired

4 Housewife ≤5 Unemployed

3) Education

1 No formal education ≤2 Primary School ≤3 Up to Form 3

4 Up to Form 7 5 Diploma ≤6 University Degree or above

4) Marital Status

1 Married 2 Single 3 Separated ≤4 Divorced

5 Widowed ≤6Co-habitate

5) Are you receiving Comprehensive Social Security Assistance(CSSA) or other financial support?

1 Yes ≤2 No

6) Monthly Salary (in HK$) (including financial support from your children and the government)

1 Less than 5000 2 5001-10000 3 10001-15000 4 15001-20000

5 20001-25000 6 25001-30000 7 30001-35000 8 35001-40000

9 40000 above ≤10 Decline to answer

7) Housing

1 Private property ≤2 Home Ownership Scheme (HOS) ≤3 Public Housing

4 Rental ≤5 Other: ________________

========================== The End. Thank you! =========================
